# Common Ingredient Profiles of Multi-Ingredient Pre-Workout Supplements

**DOI:** 10.3390/nu11020254

**Published:** 2019-01-24

**Authors:** Andrew R. Jagim, Patrick S. Harty, Clayton L. Camic

**Affiliations:** 1 Human Performance Lab, Sports Medicine, Mayo Clinic Health System, Onalaska, WI 54650, USA; 2 Exercise & Performance Nutrition Laboratory, Lindenwood University, St. Charles, MO 63301, USA; pharty@lindenwood.edu; 3 Kinesiology and Physical Education, Northern Illinois University, DeKalb, IL 60115, USA; ccamic1@niu.edu

**Keywords:** ergogenic aid, supplement, pre-workout, beta-alanine, caffeine, citrulline, creatine, strength, power, energy

## Abstract

Multi-ingredient pre-workout supplements are a popular class of dietary supplements which are purported to improve exercise performance. However, the composition of these products varies substantially between formulations, thus making comparisons challenging. Therefore, the purpose of this study was to identify a common ingredient profile of top-selling pre-workout supplements and to compare ingredient dosages to established efficacious values. The top 100 commercially available pre-workout products were analyzed for listed ingredients and amounts, if available, from the supplement facts panel. The mean ± SD number of ingredients per supplement (*n* = 100) was 18.4 ± 9.7 with 8.1 ± 9.9 of these ingredients included in a proprietary blend at undisclosed quantities. Relative prevalence and average amounts of the top ingredients amounted to: Beta-alanine (87%; 2.0 ± 0.8 g), Caffeine (86%; 254.0 ± 79.5 mg), Citrulline (71%; 4.0 ± 2.5 g), Tyrosine (63%; 348.0 ± 305.7 mg), Taurine (51%; 1.3 ± 0.6 g), and Creatine (49%; 2.1 ± 1.0 g). Nearly half (44.3%) of all ingredients were included as part of a proprietary blend with undisclosed amounts of each ingredient. The average amount of beta-alanine per serving size was below the recommended efficacious dose. The average caffeine content was near the low end for an effective relative dose for a 70 kg individual (3–6 mg·kg^−1^ of bodyweight).

## 1. Introduction

Dietary supplements represent approximately a 30-billion-dollar industry in the United States alone [1], with over 50% of US adults reporting the regular use of one or more supplements [2]. In 1994, Congress passed the Dietary Supplement Health and Education Act, which established an official definition for a dietary supplement, stating that in order for a product to be classified as a dietary supplement it must meet one or more of the following: (1) A vitamin, (2) A mineral, (3) A herb or other botanical, (4) An amino acid, (5) A dietary substance for use by man to supplement the diet by increasing the total dietary intake, or (6) A concentrate, metabolite, constituent, or extract of one of the above categories [3]. Dietary supplement manufacturers have a lot of liberty in how they design and formulate their products. Currently, there is not a pre-market approval process needed to document the efficacy and safety before new dietary ingredients can be included into a manufactured dietary supplement product, as long as they meet the aforementioned criteria. However, the Food and Drug Administration (FDA) does require a pre-market notification of a new dietary ingredient (NDI) to be submitted by the manufacturer at least 75 days prior to marketing the NDI in a supplement [4]. Additionally, the FDA has strict guidelines for supplement facts panel labeling in regards to what ingredients are required to be listed on the label and how they are presented [5], in an attempt to provide the consumer with adequate information regarding the ingredients included in the dietary supplement. Otherwise, dietary supplement manufacturers are able to formulate products at their own discretion which leads to variable ingredient profiles across the market.

Multi-ingredient pre-workout supplements (MIPS) are a specialized category of dietary supplements intended to be consumed prior to exercise that include a blend of ingredients purported to increase exercise performance [6]. A recent 2018 review found MIPS ingestion led to acute ergogenic benefits following a single dose while subsequently augmenting training adaptations when routinely ingested over time in conjunction with a training program [7]. The authors also found minimal reports of adverse events following continued supplementation. MIPS often contain a proprietary blend or matrix formula in an attempt to maintain a level of confidentiality with product formulations [8]. The FDA only mandates that the total weight of the ingredients included in the blend are listed but the individual ingredients amounts themselves do not need to be disclosed [5]. However, the ingredients need to be listed in a descending order of predominance by weight and followed by a symbol specifying that the daily values of such ingredients are not established [5]. Unfortunately, this does not provide the consumer with adequate information to determine whether an effective dose of each ingredient is included in the product or to ensure safety if they are also consuming other related dietary supplement products. For example, if an individual consumes a MIPS in addition to a multivitamin, an energy shot, or other caffeinated beverage, they could be unknowingly at risk of ingesting high levels of ingredients such as caffeine or niacin that could result in adverse events.

A variety of factors likely influence manufacturer decisions when it comes to product development of MIPS, such as physiological rationale for structure and function claims, clinically supported evidence of efficacy, cost efficiency, raw ingredient availability, patents of formulations, and taste or texture. There does appear to be a common core list of ingredients commonly found in MIPS, including caffeine, beta-alanine, creatine, citrulline, taurine, tyrosine, and B vitamins; however, not all products contain the same core ingredients, making it difficult to compare one product to another [7]. Further, MIPS are sometimes confused with energy drinks, energy shots, blood flow enhancers, or weight loss supplements as they can contain similar ingredients. Although there is some overlap of certain ingredients, the purported benefits and mechanisms of action of the respective products are different and tend to include different complimentary ingredients [9,10,11]. For example, MIPS tend to contain ingredients that are purported to improve acute exercise performance in addition to enhancing training adaptations over time such as caffeine, beta-alanine, creatine, and betaine. Conversely, energy shots and energy drinks generally only contain caffeine and a select number of vitamins or amino acids that are more targeted at increasing energy or levels of alertness and can be ingested at any time throughout the day. Furthermore, they tend to lack any additional ingredients that influence muscular performance or adaptations. Currently, there is not a well-established list of criteria that classifies a supplement as a MIPS, nor is there an accepted common ingredient profile of such a product. Therefore, the focus of the current study was to examine supplement facts panels of the top selling commercially available MIPS within the US to characterize common ingredient profiles to help establish a standard definition of a MIPS product. A secondary aim of the study was to compare the mean ingredient profile of commonly identified ingredients (if disclosed) and to evidence-based recommendations regarding an efficacious dose.

## 2. Materials and Methods

The top 100 commercially available MIPS were identified and compiled from a commercial retail website (www.bodybuilding.com) as of October 2018. Independent sales data were not available, so the included MIPS products were identified after filtering the searched products using a “best-selling” criteria as previously used by Desbrow and colleagues [12]. For the purpose of this study, a MIPS product must have met the following criteria to be classified as a pre-workout supplement and included in the analysis: (A) Marketed as a pre-workout supplement; (B) Recommended to be ingested prior to exercise; and containing at least three of the following ingredients: (1) Caffeine, (2) Beta-alanine, (3) Citrulline, (4) Niacin, (5) Creatine, (6) Taurine, (7) Tyrosine, (8) Arginine, (9) Branched chain amino acids, (10) Choline Bitartrate, or (11) Betaine. These criteria were used to create distinction between MIPS and other similar products that may be marketed as a pre-workout supplement but are more similar to strictly blood flow enhancers or energy shots. Two research staff members independently analyzed each supplement facts label to compile listed ingredients and corresponding amounts, if available, for each product. Special notation was used for ingredients that were listed on the product label as part of a propriety blend and therefore specific ingredient amounts were not available. Only ingredients included in the nutrition facts and supplement facts sections of the label were included. Thus, ingredients listed as “other ingredients”, such as preservatives, artificial flavoring agents, and food coloring, were not included in the analysis. Because the information used for analysis is publicly available, approval from an Institutional Review Board was not required.

### Statistical Analysis

All data are presented as means ± SD. Basic descriptive and frequency analysis was performed for the included ingredients across each product to create a common MIPS ingredient profile. Means and standard deviations were reported for each ingredient explicitly listed on the supplement facts label. Frequency analysis was used to determine the relative prevalence of listed and unlisted ingredients within the dataset as well as the prevalence of supplements included in the analysis which contained an ergogenic amount of key ingredients. Linear regression analysis was employed to determine the relationship between the included amount of key ingredients and total serving size of each supplement. Correlation coefficients were interpreted as weak (r ≤ 0.35), moderate (0.36 ≤ r ≤ 0.67), or strong (0.68 ≤ r ≤ 0.99) [13]. An alpha level of 0.05 was used for all statistical determinations.

## 3. Results

### 3.1. Ingredient and Proprietary Blends

The mean ± SD number of ingredients per pre-workout supplement (*n* = 100) was 18.4 ± 9.7 with 8.1 ± 9.9 of these ingredients included in a proprietary blend at undisclosed quantities. The percentage of total ingredients included in a proprietary blend for all 100 supplements was 44.3%. In addition, 58/100 supplements included at least one proprietary blend with 14.0 ± 9.2 ingredients, or 63.8 ± 24.4% of all ingredients, at undisclosed quantities.

### 3.2. Specific Ingredients

Table 1 provides the prevalence (%) of specific ingredients included in the 100 pre-workout supplements for both undisclosed and listed amounts, the mean ± SD quantities of ingredients that were listed on the label, the suggested ergogenic quantity of each ingredient, and the prevalence (%) of supplements that contained each ingredient at this suggested ergogenic level.

### 3.3. Ingredient Amounts versus Serving Size

**Figure 1:**
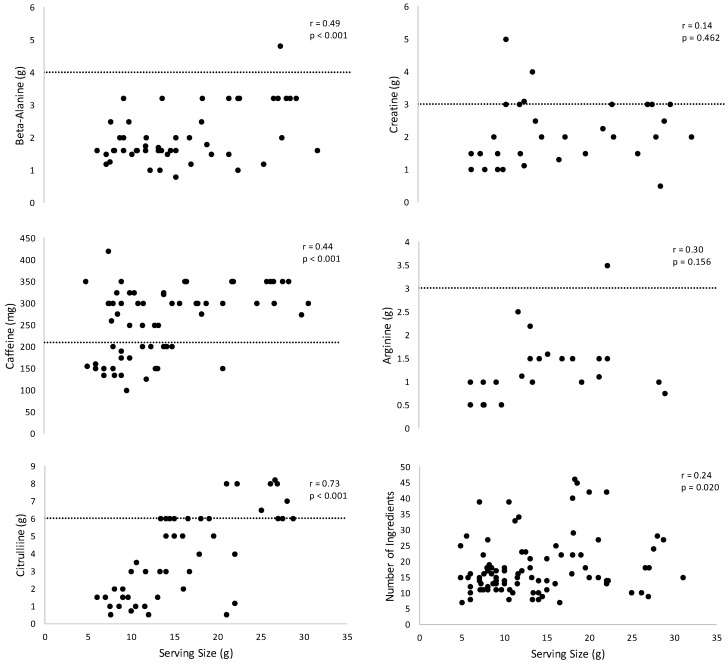
The relationships for beta-alanine (*n* = 56), caffeine (*n* = 70), citrulline (*n* = 47), creatine (*n* = 31), arginine (*n* = 24), and number of ingredients (*n* = 94) versus serving size for the pre-workout supplements. Dashed line represents the minimum suggested ergogenic quantity.

The results of the simple linear regression analyses indicated there were significant relationships for amount of caffeine (r = 0.44, *p* < 0.001, *n* = 70), beta-alanine (r = 0.49, *p* < 0.001, *n* = 56), citrulline (r = 0.73, *p* < 0.001, *n* = 47), and number of ingredients (r = 0.24, *p* = 0.020, *n* = 94) plotted across serving size (Figure 1). There were no significant relationships, however, for creatine (r = 0.14, *p* = 0.462, *n* = 31) or arginine (r = 0.30, *p* = 0.156, *n* = 24) versus serving size (Figure 1).

## 4. Discussion

The purpose of this investigation was to determine a typical ingredient profile of best-selling MIPS products by establishing a standard definition of a MIPS used to identify such products in the analysis and to determine the prevalence of best-selling MIPS formulations containing efficacious doses of well-supported ergogenic ingredients. Based upon the results of the current evaluation, the most popular MIPS ingredients appear to be beta-alanine, caffeine, citrulline, tyrosine, taurine, creatine, and niacin, as each of these ingredients appeared in at least 48% of the top 100 selling MIPS products (Table 1). Average listed amounts and overall relative prevalence of these ingredients in the sample amounted to the following: Beta-alanine (prevalence: 87%; average listed amount: 2.0 ± 0.8 g), Caffeine (prevalence: 86%; average listed amount: 254.0 ± 79.5 mg), Citrulline (prevalence: 71%; average listed amount: 4.0 ± 2.5 g), Tyrosine (prevalence: 63%; average listed amount: 348.0 ± 305.7 mg), Taurine (prevalence: 51%, average listed amount: 1.3 ± 0.6 g), Creatine (prevalence: 49%, average listed amount: 2.1 ± 1.0 g), and Niacin (prevalence: 48%, average listed amount: 25.8 ± 15.2 mg). These results are somewhat similar to the conclusions of Eudy and colleagues [8], who identified caffeine, creatine, beta-alanine, and taurine as typical ingredients in pre-workout supplements in a 2013 review. However, the researchers classified several common energy drinks as pre-workout supplements, thus skewing the typical ingredient profile of the included products. It is important to note that the average listed caffeine content of the products included in the current investigation (254.0 ± 79.5 mg) was in accordance with the findings of a previous study by Desbrow et al. [12], which investigated the caffeine content in top selling MIPS products in Australia and found average caffeine content per serving to be 100–390 mg. Interestingly, the authors noted a high degree of variation between the caffeine dose presented on the label and the actual caffeine content of a given product as determined by high performance liquid chromatography. There was also a degree of variability from one batch to another.

It is worth noting that the average amounts of certain ingredients do not appear to align with the clinically-supported dosing recommendations for beta-alanine [14], caffeine [15], citrulline [16], creatine [17], or arginine [18], as depicted in Figure 1. Because pre-workout supplements provide a variety of ingredients to the consumer in a single formulation, it is vital that ingredients be included at efficacious doses as this ultimately determines the long-term ergogenic potential. However, the high prevalence of proprietary blends in this sample limited the assessment of dosing efficacy to ingredients with explicit amounts listed on a given supplement facts label. Nonetheless, the analysis of listed ingredient amounts provides valuable information regarding the potential of the well-supported ergogenic ingredients included in these supplements to confer any substantial benefit. For example, the average creatine content of the included MIPS products was 2.1 ± 1.0 g, which is below the minimum dose of at least 3 g·day^−1^ and the standard recommended daily dose of 5 g·day^−1^ that has been supported within the literature [17]. Furthermore, only 29% of MIPS products in this investigation listed creatine in amounts greater than or equal to the minimum ergogenic quantity. Similarly, the average listed beta-alanine content was 2.0 ± 0.8 g, which is also below the minimum ergogenic dose of 4 g·day^−1^ [14]. Interestingly, while the amount of beta-alanine was listed in 57 of the included products, only one of these products contained a dose greater than the threshold of efficacy. Clearly, consumers using these products who wish to increase intramuscular carnosine levels and subsequently improve intramuscular buffering capacity will need to consume beta-alanine from other supplemental sources. In contrast to several of the earlier ingredients, the average caffeine content of the included MIPS was 254 ± 7.5 mg, which is above the 210 mg required to meet the lower threshold for a recommended dose (3–6 mg·kg^−1^ of bodyweight) reported within the literature [15]. However, this recommendation is based on a 3 mg·kg^−1^ dose for a 70 kg individual. Thus, while 77.2% of the included formulations contained more than 210 mg of caffeine, these formulations may not confer ergogenic effects in individuals with a body weight greater than 70 kg, which may often be the case as MIPS products are typically marketed towards individuals engaged in resistance training or bodybuilding practices who are likely larger than 70 kg. Additionally, it is important to note that because most MIPS products are marketed towards this population and previous reviews [19,20,21] indicate that a caffeine intake closer to 6 mg·kg^−1^ of bodyweight is likely needed to elicit an ergogenic benefit during resistance exercise, the average caffeine content in MIPS may also be insufficient for those consuming them for strength or power-related benefits. Because the minimum suggested doses for several of these ingredients were rarely met, it is clear that many MIPS formulations are inadequate sources of ergogenic ingredients, such as beta-alanine, citrulline, and creatine, and may not deliver enough caffeine to provide performance benefits in larger individuals.

The high prevalence of proprietary blends within the sample (58% of all MIPS products) has significant implications for consumers, as each proprietary blend contained 14.0 ± 9.2 ingredients (63.8 ± 24.4% of all listed ingredients) on average. As mentioned previously, while FDA regulations mandate that all ingredients in a blend be listed in descending order of predominance by weight, it is difficult to determine whether individual ingredients in a proprietary blend are included in doses sufficient to elicit ergogenic effects [7]. However, the results of this study suggest total serving size of a given MIPS product may serve as a potential indicator of supplement efficacy, as several ingredients in the sample were shown to be positively correlated with serving size (Figure 1). For example, moderate to high correlations were found between total MIPS sample size and the following ingredients: caffeine (r = 0.44, *p* < 0.001, *n* = 70), beta-alanine (r = 0.49, *p* < 0.001, *n* = 56), and citrulline (r = 0.73, *p* < 0.001, *n* = 47). However, no significant relationships were found between total supplement serving size and creatine or arginine. Though the relationship between serving size and amount of key ingredients could only be determined in open-label products, serving size may also serve as a key metric when evaluating the efficacy of MIPS products with a high percentage of ingredients contained in a proprietary blend. For example, a MIPS with a serving size of 8 g will not contain both the 6 g minimum ergogenic dose of citrulline [16] and the 4 g minimum dose of beta-alanine [14]. Thus, we recommend that supplement consumers consider total serving size when making purchasing decisions, as products with a larger serving size likely contain greater doses of several key ergogenic ingredients.

A concerning trend identified by this investigation was the average listed vitamin B3 (niacin) content in the included products (25.8 ± 15.2 mg), which approached the tolerable upper intake level (UL) of 35 mg·day^−1^ [22]. To date, several investigations have linked the consumption of niacin-containing energy products with acute hepatitis [23,24] and liver failure [25] in otherwise-healthy adults. Though niacin content was explicitly listed on all MIPS which contained it as an ingredient, it is possible that consumers may not fully understand the potential hazard of consuming multiple servings of MIPS or combining MIPS with other niacin-containing products such as energy shots or energy drinks [10]. Similarly, consuming multiple servings of MIPS or combining MIPS with other caffeine-containing substances may place consumers at risk of adverse events resulting from excess caffeine consumption, including tachycardia, heart palpitations, headache, anxiety, and sleep disturbance [26,27]. However, the average caffeine content in the included MIPS products was found to be 254 ± 79.5 mg, which is well below the 400 mg·day^−1^ (6 mg·kg^−1^) threshold of consumption suggested by Nawrot and colleagues [28] in a seminal review. Thus, provided that MIPS consumers keep daily niacin and caffeine intake within acceptable limits, the risk of adverse events resulting from these substances will likely be mitigated.

A limitation of this investigation is that presented values are based off the provided supplement facts panel from the manufacturer and may not reflect actual ingredient amounts contained within the product as it is off the shelf. As highlighted by Desbrow et al. [12], select ingredient amounts may vary from batch to batch or within a single batch and may not reflect posted ingredient amounts on the provided supplement facts label. Therefore, results of this study should be interpreted within the appropriate context of profiling supplement ingredients from supplement facts labels and not laboratory-based methods of assessing actual ingredients contained in these products.

## 5. Conclusions

By establishing a common ingredient profile and more standard definition of a MIPS, future research can now better investigate the efficacy, and more importantly, the safety of these products by creating a level of distinction between MIPS and other related products. The findings of the present investigation indicated that beta-alanine and citrulline are the most common ingredients found in multi-ingredient pre-workout supplements. The average amount of beta-alanine per serving size falls well below the recommended efficacious dose. The average dose of caffeine is near the low end of an effective relative dose (3–6 mg·kg^−1^ of bodyweight) for a 70 kg individual at 3.6 mg·kg^−1^ of bodyweight. Additionally, nearly half of the ingredients in MIPS are manufactured as part of a proprietary blend with undisclosed amounts of each ingredient. It may be in the best interest of the consumer to select a MIPS product that discloses all ingredient amounts and contains appropriate dosages of each ingredient. Consumers may also want to pay close attention to specific ingredients that may be under-dosed (beta-alanine and creatine in particular), as they may want to supplement with other products in order to optimize the associated benefits. Further, it is advisable to reduce ingestion of other caffeine or niacin containing products to minimize the risk of any adverse events from consuming excess amounts. Future research should examine how individuals are utilizing these products, particularly if they are being combined with other related products, in an attempt to identify any safety concerns. Additionally, more information is needed regarding the purity and quality of MIPS products, specifically ones that contain proprietary blends, so that individuals know exactly what they are consuming with each serving.

## Figures and Tables

**Table 1:** Prevalence and quantities of ingredients included in the bestselling pre-workout supplements (*n* = 100). MIPS: Multi-ingredient pre-workout supplements.

Ingredient	Overall Prevalence (%)	Prevalence in Undisclosed Quantity (%)	Prevalence in Listed Quantity (%)	Mean ± SD Listed Quantity	Suggested Ergogenic Quantity	Prevalence (%) of MIPS ≥ Ergogenic Quantity
Beta-Alanine (g)	87	30	57	2.0 ± 0.8	4.0	1 (1.8%)
Caffeine (mg)	86	15	71	254.0 ± 79.5	210.0	44 (77.2%)
Citrulline (g)	71	23	48	4.0 ± 2.5	6.0	18 (37.5%)
Tyrosine (mg)	63	31	32	348.0 ± 305.7		
Taurine (g)	51	22	29	1.3 ± 0.6		
Creatine (g)	49	18	31	2.1 ± 1.0	3.0	9 (29.0%)
Niacin (mg)	48	0	48	25.8 ± 15.2		
Arginine (g)	46	21	25	1.3 ± 0.7	3.0	1 (4.0%)
Vitamin B12 (μg)	45	0	45	121.7 ± 227.2		
Betaine (g)	33	8	25	1.9 ± 0.8		
Choline Bitartrate (mg)	30	13	17	376.5 ± 243.0		
Theanine (mg)	24	6	18	121.6 ± 83.9		
BCAAs (g)	22	11	11	3.2 ± 1.9		
Carnitine (mg)	19	8	11	686.4 ± 634.4		
Yohimbe (mg)	15	5	10	19.2 ± 11.6		
Beet Root Extract (mg)	6	3	3	400.5 ± 173.2		
